# Efficacy of Acupuncture for Health Conditions in Children: A Review

**DOI:** 10.1100/tsw.2008.86

**Published:** 2008-07-13

**Authors:** Jean Libonate, Subhadra Evans, Jennie C. I. Tsao

**Affiliations:** ^1^ Emperor's College of Traditional Oriental Medicine, Santa Monica, CA, USA; ^2^ Pediatric Pain Program, Department of Pediatrics, David Geffen School of Medicine, University of California Los Angeles, USA

**Keywords:** children, human development, acupuncture, holistic health

## Abstract

Acupuncture has been used to treat a variety of childhood problems; however, the efficacy and safety of pediatric acupuncture remains unclear. This article reviews the existing empirical literature relating to the use of acupuncture for medical conditions in children. A systematic search of the literature revealed that acupuncture has been used to treat five main conditions in children, including pain, nocturnal enuresis, postoperative nausea/vomiting, laryngospasm/stridor, and neurological disorders. Despite a number of methodological issues, including limited sample sizes, lack of randomization, and inappropriate control groups, it is concluded that acupuncture represents a promising intervention for a variety of pediatric health conditions. To further address the safety, effectiveness, and acceptability of acupuncture in children, large-scale randomized controlled trials are needed.

## INTRODUCTION

Traditional Chinese medicine teaches that an energy force called Qi (pronounced chee) flows throughout the body by way of channels or meridians that, if blocked, cause imbalance or sickness. Acupuncture is intended to restore the flow of Qi through the insertion of needles into points along energy pathways (meridians) in the body. The needles help to stimulate and balance the energy flow. Usually, needles are inserted into the skin from ¼ to 1 in. deep. Patients may report heaviness, distention, warmth, or an electric sensation either around the needle or traveling up or down the energy pathway; other patients report no sensation at all. Within conventional Western medicine, the exact analgesic mechanisms of acupuncture are currently unknown. However, it is likely that the body’s nervous system, neurotransmitters, and endogenous substances are involved in needle stimulation.

The use of acupuncture and oriental medicine is gaining popularity in the U.S.[1]. As these approaches become more familiar and comfortable to the adult population, parents are more likely to take their children to an acupuncturist. While the pediatric population as a whole may report minimal use of acupuncture[2], there are pockets of more concentrated use. For example, one suburban pediatric practice in down-state New York reported that approximately 34% of their patients use complementary and alternative medicine (CAM) at any time[3]. However, unless there is evidence to support the safety and efficacy of acupuncture for children, pediatricians may not advocate its use. It is the intention of this review to evaluate current research on acupuncture use for pediatric populations in order to guide pediatricians and other health professionals who are considering using or recommending acupuncture.

## METHODS

This review is limited to research that focuses on children through age 20. Given that acupuncture research is limited, work from all countries was reviewed, providing that the studies were translated into English and published in peer-reviewed journals. Studies that used randomized controlled trials (RCTs), controlled trials, convenience samples, or repeated measure designs were included. Case studies were excluded, as were studies that included acupuncture as only part of treatment (such as herbal supplements along with acupuncture). Research from 1990 onwards is included in the review, due to the scant research before that time, only some of which is in English and easily obtainable.

For the purposes of the review, acupuncture was defined as use of needles, bleeding, laser, or electrical stimulation and pressure to acupuncture points. Acupressure is commonly used for children to alleviate their potential fear of needles. However, previous research reported relatively few problems with needles in children[1], with another acupuncture study finding that over 90% of children completed the protocol, indicating that fear of needles was not especially problematic[4]. Studies that included herbal administration, cupping, tui na, or other therapies that involved more than needles, pressure, bleeding, laser, or electrical stimulation to acupuncture points were excluded. The following computerized databases were searched for available literature using the keywords “pediatric”, “acupuncture”, and “child”: PubMed, Ovid, Cinahl, and Psycinfo. The literature search revealed that acupuncture has been used for a variety of complaints in children, including pain, nocturnal enuresis, nausea/vomiting, laryngospasm/stridor, and neurological disorders. A sixth group of miscellaneous studies was also identified. The findings are discussed around these six main categories below and summarized in Tables 1–6.

### Pain Management Studies

Two pediatric acupuncture studies were found that pertained specifically to pain management (see Table 1). One RCT used acupuncture for migraine headaches and incorporated the use of blood analysis for biological markers[5]. Treatment for the true acupuncture group involved inserting needles subdermally; for the placebo acupuncture group, the same-sized needle was inserted into the stratum corneum. Blood samples were drawn for panopioid activity before the first treatment, and after the fifth and tenth treatments and compared to 10 children without migraines. Patients in the acupuncture groups completed migraine frequency/intensity questionnaires prior, during, and following the study. The true acupuncture group reported a significant clinical reduction in frequency and intensity of migraines; the sham group had no significant change. In addition, the true acupuncture group demonstrated a significant change in plasma panopioid activity and an elevation of β-endorphin immunoreactivity. Strengths of the study included double blinding, thorough assessment of functioning before treatment, and use of a randomized controlled design; however, the small sample size limits the utility of the findings. Another concern is that the authors did not reveal the exact points used for treatment.

The second study explored acupuncture and hypnosis for a pediatric pain management clinic[4]. Children at a tertiary clinic specializing in the treatment of pediatric pain received six weekly sessions of individualized acupuncture, with a simultaneous 20-min hypnosis session. Parent and child reports revealed a significant reduction in pain and improvement in daily functioning following treatment[4]. The study supports the feasibility of combining acupuncture and hypnotherapy treatments. However, only children with select chronic pain diagnoses were included. This limits generalizability to other conditions and given the lack of a control group, also limits conclusions regarding the treatment’s efficacy.

**TABLE 1**
**Pain Management**

| Study | Design | Group Type | Subjects | # of Tx. | Outcome Measures | Results |
|---|---|---|---|---|---|---|
| Pintov 1997 (Israel) | RCT<br>Single blind | True acupuncture<br>Placebo acupuncture<br>Healthy controls | n = 10 (each group)<br>7–15 years<br>59% female | 10 weekly | Pain<br>Opioid blood assay | _True acupuncture_:<br>↓Frequency, intensity of headaches<br>↑Panopioid plasma<br>↑β-endorphin level |
| Zeltzer 2002 (U.S.) | Convenience | Acupuncture with hypnosis | n = 31<br>6–18 years<br>61% female | 6 weekly | Patient and parent pain reports | Combining acupuncture with hypnosis is feasible. |

## Postoperative Nausea and Vomiting

Acupuncture has been used to treat postoperative nausea and vomiting (PONV) for various surgical procedures, as presented in Table 2. A group of studies using acupuncture for nausea in tonsillectomy produced mixed findings. In one large study, 100 children undergoing tonsillectomy were randomly assigned to an acupressure/acupuncture group or a sham placebo group[6]. In the treatment group, acupressure was applied, followed by inserting an intradermal needle into the same point after anesthesia induction. The placebo group had bands placed at the same location. The study failed to show a statistically significant antiemetic for acupuncture. The intervention was performed by four anesthesiologists who were trained by a licensed acupuncturist; however, it is questionable whether this training was a valid substitute for treatment by a licensed acupuncturist. Another smaller study also reported no difference in vomiting between an acupuncture and an unspecified control group[7]. The findings of this study are inconclusive however, as it is not known what the control condition entailed.

A larger study of children undergoing tonsillectomy with or without adenoidectomy included three groups: acupuncture with electrical stimulation, sham acupuncture with stimulation, and no acupuncture[8]. The no acupuncture controls had insulated wires attached to arm covers with the electrostimulation machine turned on to simulate treatment. Nausea was significantly reduced in the acupuncture group compared to the sham and no acupuncture groups. In addition, the sham group had an increased incidence of rescue therapy and vomited earlier than the other groups. Similar positive findings have been reported by Kabalak and colleagues[9], who found that vomiting was significantly reduced in children undergoing tonsillectomy treated with electrical acupoint stimulation compared to a no treatment control group. The benefits of the acupoint treatment were similar to those seen in a standard care medication group, and significantly fewer side effects were reported in the acupoint group compared to the treatment controls. The findings suggest that electrical acupoint stimulation may be at least as effective as standard care using medication, with fewer unwanted side effects.

Four studies have used a variation of acupuncture techniques for nausea following strabismus surgery. In the earliest of the studies, acupressure administered by commercially available wrist bands with plastic studs to stimulate the P6 point were not found to be any different to sham acupressure bands without studs in preventing postoperative vomiting[10]. Given the likely size difference between younger vs. older children in this study (the age range was 3–12 years), it is possible that the studs varied widely in pressure location and not all children had the correct point activated. A further RCT for vomiting following strabismus surgery compared Korean hand acupressure (as administered by a professional) to a placebo involving tape placed in the same location[11]. The acupressure group showed a significantly lower incidence of vomiting in the postoperative period compared to the placebo group. A study using

**TABLE 2**
**Postoperative Nausea and Vomiting**

| Study | Design | Group Type | Subjects | # of Tx. | Outcome Measures | Results |
|---|---|---|---|---|---|---|
| Lewis 1991 (U.K) | RCT Double blind | Acupressure<br>Banded placebo | n = 33<br>n = 31<br>3–12 years<br>38% female | Continuously from 1 h presurgery | Vomiting during first 24 h | No differences |
| Yentis 1991 (Canada) | RCT Double blind | Acupuncture<br>Unspecified control | n = 23<br>n = 22<br>2–11 years<br>? female | 1 Tx after anesthesia | Vomiting | No differences |
| Yentis 1992 (Canada) | RCT Double blind | Acupuncture<br>Meds<br>Acupuncture+ meds | n = 30 (each group)<br>2–11 years<br>? female | Single tx before surgery<br>i.v. droperidol | Vomiting/retching in recovery room, restlessness | No differences in vomiting<br>↓Restlessness in acupuncture only group |
| Schlager 1998 (Austria) | RCT Double blind | Laser acupoint<br>Placebo laser stimulation | n = 20<br>n = 20<br>3–12 years<br>53% female | 2 Treatments: each 15 min before and after surgery | Vomiting in first 24 h postop | ↓Vomiting in laser acupoint–stimulated group |
| Shenkman 1999 (USA) | RCT | Acupressure (intradermal needles)<br>Sham acupressure (intradermal needles) | n = 47<br>n = 53<br>2–12 years<br>50% female | Continuous tx preop to intradermal needles after anesthesia | Retching and vomiting postop | No significant differences |
| Schlager 2000 (Austria) | RCT Double blind | Acupressure<br>Placebo | n = 25 (each group)<br>3–12 years<br>50% female | Single treatment 30 min preop through 24 h | Vomiting in first 24 h postop | ↓Vomiting for acupressure group |
| Somri 2001 (Israel) | RCT | Acupuncture<br>i.v. Med.<br>Control (i.v. fluid only) | n = 30 (each group)<br>4–12 years<br>46% female | 15 min<br>20-min infusion<br>20-min infusion | Vomiting, admissions, parental satisfaction. | ↓Vomiting<br>↑Satisfaction in acupuncture and i.v. med groups |
| Rusy 2002 (USA) | RCT Double blind | Electroacupuncture<br>Sham control<br>No acupuncture | n = 40 (each group)<br>4–18 years<br>48% female | Single tx after tonsillectomy | Nausea, vomiting, rescue therapy in first 24 h postop | ↓Nausea for electroacupuncture, vomiting not significantly different |
| Wang 2002 (USA) | RCT Double blind | Acupoint injections<br>Med. + sham acupuncture<br>Sham acupoint<br>Sham acupuncture | n = 50<br>n = 49<br>n = 43<br>n = 45<br>7–16 years<br>44% female | i.v. Meds + acupoint injection or s.c. acupuncture at end of surgery | Nausea and vomiting early and late postop | ↓PONV for acupoint injection and i.v. med groups over sham groups |

*Table 2 continues*

**TABLE 2 (continued)**

| Study | Design | Group Type | Subjects | # of Tx. | Outcome Measures | Results |
|---|---|---|---|---|---|---|
| Butkovic 2005 Croatia | RCT | Laser acupuncture<br>Meds + sham laser<br>Sham laser | n = 40 (each group)<br>6–9 years<br>69% female | Single tx before anesthesia | Vomiting and retching | ↓Vomiting in both tx groups |
| Kabalak 2005 (Turkey) | RCT | Acustimulation<br>Meds<br>No treatment | n = 30 (each group)<br>4–12 years<br>48% female | Pre- and postsurgery | Nausea, drowsiness, parent satisfaction | ↓Nausea in both tx groups;<br>↓Side effects for acu-stimulation |
| Reindl 2006 (Germany) | Randomized crossover | Meds+ acupuncture<br>Meds | n = 11<br>10–16 years<br>64% female | First day of chemo-therapy + patient request | Vomiting, nausea, weight, meds | ↑Alertness<br>↓Meds for acupuncture |

low-level diode laser stimulation vs. placebo (inactivated laser) administered before and after surgery reported significantly less vomiting in the treatment group[12]. A further study for PONV following strabismus surgery reported no differences between patients who received acupuncture alone, medication alone, or acupuncture plus medication in incidence of vomiting[7]. However, acupuncture alone was associated with less restlessness than the other groups, indicating that acupuncture may be helpful for children following tonsillectomy. Since there were no differences in vomiting, acupuncture may have reduced arousal or had some other unspecified effects in this study; it did not appear to be differentially effective in preventing vomiting.

Further evidence that acupuncture is associated with reduced harmful effects compared to medication was revealed in a study examining acupuncture for chemotherapy-induced nausea and vomiting[13]. Here, an acupuncture alone and an acupuncture plus medication group had similar PONV outcomes; however, the need for additional postoperative nausea medication was lower in the acupuncture only group, which also reported decreased drowsiness. The conclusions of this study are tempered by the small sample size and difficulty comparing groups since both were initially administered antiemetics. A study comparing acupuncture to an antiemetic medication (ondansetron) and a placebo in 90 children undergoing surgery for dental restoration revealed a significant decrease in emetic episodes between the acupuncture and ondansetron groups compared to the placebo group[14]. Satisfaction rates were also higher for the acupuncture and ondansetron groups, indicating that acupuncture is as effective as medication for treating postsurgery nausea.

In the largest pediatric double-blind RCT, acupoint injections were compared to droperidol for control of PONV for general outpatient surgery[15]. Children were randomly assigned to acupoint, droperidol, and sham point groups. In the early postoperative period, nausea and vomiting were significantly lower in the acupoint group as compared with the sham groups, but there was no significant difference between the acupoint and droperidol groups. Earlier differences disappeared in the late postoperative period, indicating that benefits were limited to directly after the procedure. These findings further suggest that acupuncture may have similar antinausea effects to antiemetic medication. Similar findings emerged for another large RCT using acupuncture for a variety of surgical procedures[16]. In this study, laser acupuncture was found to have similar benefits to metoclopramide compared to a control group using sham lasers. Both treatments resulted in reduced vomiting/retching 2 h postsurgery; treated children were also less likely to require rescue medication. Although the study has a number of limitations, including lack of blinding and unclear measurement procedures, the findings present further evidence of the efficacy of acupuncture to treat PONV.

On the whole, these studies indicate that acupuncture can be effective in reducing PONV, and may be superior to current antiemetic medications given that a number of studies have reported reduced side effects for acupuncture compared to medication. These conclusions are strengthened by the use of randomization, double blinding, and substantial sample sizes for most studies. If repeated studies show similar results, acupuncture should be considered before use of pharmaceuticals for nausea and vomiting. However, there were wide variations in treatment applications with electrical stimulation, acupressure, intradermal acupuncture, laser acupuncture, and acupoint injections all employed. Further research should clarify which specific method is superior.

## Nocturnal Enuresis

Based on epidemiological studies, nocturnal enuresis is a common problem among children[17]. Use of acupuncture for nocturnal enuresis has been the subject of a number of studies (see Table 3). The majority of pediatric nocturnal enuresis subjects were male, consistent with the higher prevalence of the condition in boys[18]. A study by Serel[19] used acupuncture for patients who did not respond to pharmacological treatments. Frequency of acupuncture treatments was tapered as the patients responded by increasing their number of dry nights. Within 6 months, 86% of patients were completely dry and 4% were dry for at least 80% of nights. At a 13-month follow-up, 40 patients were completely dry, while three patients were not helped with acupuncture. In a smaller study of 15 patients, acupuncture was associated with increased bladder capacity; three patients were considered cured after 1 week of treatment and seven cured after 2 months[20]. Although small and lacking a control group, this study suggests that acupuncture may treat nocturnal enuresis by increasing bladder capacity.

In the first of two prospective randomized trials[21], 40 children with nocturnal enuresis were treated with laser acupuncture or desmopressin. In a follow-up period of at least 6 months after treatment, 13 of 20 children in the laser acupuncture group were still dry. Response rates showed no significant differences between groups, indicating that laser acupuncture is as effective as desmopressin. Apart from the lack of a placebo control group, a further limitation of the study is that the authors did not report wavelength data, making replication in future studies and a critique of the points stimulated difficult. The second prospective randomized trial[22] studied the effects of acupressure vs. oxybutinin. Parents of 24 children were taught to provide daily acupressure treatments. Trends indicated increased success in the acupressure group, but not to significant levels. Limitations of the study include small sample size and an unclear methodology, in particular how response was defined and measured.

In a placebo-controlled crossover study[23], Korean hand acupuncture with an electromagnetic (E-beam) machine was used to determine its safety and efficacy for children with nocturnal enuresis. Patients in the acupuncture group received treatments thought to activate kidney, bladder, and cerebral functioning. After receiving five consecutive treatments with the real or placebo cords, the groups switched for five additional treatments. Patients and parents were asked to report number of dry nights directly post-treatment and 3 months post-treatment. Results indicated improvement in both groups at the end of the first and second periods. In the first period, improvements in both groups were significant; in the second period, improvement was significant only for the real treatment group. This study illustrates the possibility of spontaneous resolution of enuresis, although only the acupuncture treatment was associated with long-term gain.

Overall, acupuncture appears to have a beneficial effect on pediatric nocturnal enuresis. However, samples were generally small and varied procedures were studied, including acupuncture, acupressure, laser acupuncture, and E-beam Korean hand therapy, as well as variations in points used. The only RCT utilized a crossover model[23] and it is possible that insufficient time was given for the effects of each treatment period to “wash out” before switching treatments. The studies comparing acupuncture with established medications demonstrate that acupuncture is at least as equally effective as medication,

**TABLE 3**
**Nocturnal Enuresis**

| Study | Design | Group Type | Subjects | # of Tx. | Outcome Measures | Results |
|---|---|---|---|---|---|---|
| Radmayr 2001 (Austria) | RT (no control) | Laser acupuncture<br>Desmopressin | n = 20 (each group)<br>5–16 years<br>22% female | Intranasal x 3 months<br>10-15 (30 s) sessions | Number of dry nights | No significant difference between groups |
| Serel 2001 (Turkey) | Convenience | Acupuncture | n = 50<br>9–18 years<br>34% female | Daily x 30 minute x 10 consecutive days in a month | Number of dry nights | 86% of patients completely dry at least 80% of nights within 6 months |
| Honjo 2002 (Japan) | Convenience | Acupuncture | n = 15<br>6–18 years<br>33% female | 4 weekly | Frequency of enuresis<br>Bladder capacity | 46% improvement in 2 months<br>Significant increase in bladder capacity in all patients |
| Jodorkovosky 2003 (USA) | RCT<br>Double blind<br>Crossover | Electromagnetic<br>Alternating with placebo | n = 11<br>n = 15<br>5–13 years<br>46% female | Biweekly; five treatments per study period | Number of dry nights | Both groups improved; degree of improvement higher for electromagnetic acupuncture |
| Yuksek 2003 (Turkey) | RT (no control) | Acupressure massage by parents<br>Oxybutinin | n = 12 (each group)<br>4–13 years<br>58% female | 5 s per point per day<br>Daily dose | Incidence of bed wetting | Trends showing improvement for acupressure compared to meds |

although the results would have been strengthened with a placebo control group and blinding to treatment group. None of the studies reported negative side effects when using acupuncture. Based on these promising results and the magnitude of the problem, larger-scale research is warranted. Should further, well-designed trials establish acupuncture to be as equally effective as medication, parents and children should be given the option to pursue acupuncture as a nonpharmaceutical treatment for nocturnal enuresis.

## Laryngospasm/Stridor

Two studies (see Table 4) addressed laryngeal complications in children post–tracheal extubation. While their specific diagnostic criteria differed slightly, both studies used bloodletting at acupuncture point Shao Shang to treat or prevent complications. The bleeding technique is equivalent to a finger prick for blood work, drawing a few drops of blood. In traditional Chinese medicine, bleeding LU-11 is indicated for treatment of laryngeal conditions such as sore throat.

The first RCT[24] compared acupuncture to standard care for postextubation laryngospasm (i.e., inspiratory stridor, total closure of vocal cords, or cyanosis). Children aged 1–10 years were intubated with cuffed tubes and anesthesia was maintained with isoflurane. A significantly higher proportion of children in the control group developed laryngospasm compared to the acupuncture group. The second study[25] examined the same acupuncture bloodletting technique for the prevention of stridor after tracheal extubation

**TABLE 4**
**Laryngospasm/Stridor Studies**

| Study | Design | Group Type | Subjects | # of Tx. | Outcome Measures | Results |
|---|---|---|---|---|---|---|
| Lee 1998 (Taiwan) | RCT Single blind | Acupuncture No tx control | n = 38 (each group) 1–10 years 32% female | Single bloodletting postop | Incidence of laryngo-spasm postop | Incidence = 5.3% in treatment group; 23.7% in controls |
| Saghaei 2001 (Iran) | RCT Double blind | Acupuncture No tx control | n = 30 (each group) 1–4 years 30% female | Single bloodletting postop | Incidence of stridor postop | Incidence = 33% in treatment group; 3% in controls |

in a younger group of children aged 1–4 years. This study used halothane for anesthesia maintenance. The treatment group received bloodletting acupuncture when swallowing occurred, before extubation. An independent observer, blinded to the groups, evaluated the patients once they were released from the operating room. The incidence of stridor was significantly higher in the acupuncture group compared to the standard care control group, a reversal from the findings in the first study. The variation in methods used in these studies may have accounted for the different results. It is possible that the halothane anesthetic agent produced a lighter, shorter-lasting effect, and the children aroused faster with this agent, rendering the needle prick as more of an irritant. There is also a difference in age ranges between the studies that may have contributed to the divergent findings. It is possible that bloodletting is an ineffective method for younger children. Since the incidence of laryngospasm following halothane anesthesia is relatively low[24], there may not be justification to replicate the study with halothane. If conventional anesthetic agents are improving to the point of significantly reduced complications, the additive effect of acupuncture may be diminished.

## Neurological Disorders

A number of different neurological conditions have been the focus of the next group of studies presented in Table 5. A convenience study[25] examined the effects of acupuncture on skin temperature for children with major neurological disorders and cold feet. Four children had cerebral palsy, two had incompletely diagnosed neurological symptoms, and one had Smith-Lemli-Opitz (SLO) syndrome. Children received two treatment courses. Basal skin temperature was monitored prior to and immediately after each treatment, and at 1- and 3-month follow-ups. The results showed momentary rise of skin temperature for four children, a cumulative effect of increased skin temperature for two children, and one (with SLO syndrome) had no benefit[25]. Future research should study a larger and more carefully detailed selection of children to clarify the effects of acupuncture on skin temperature.

In a primarily qualitative study of the effects of acupuncture compared with osteopathy and a wait-list control for cerebral palsy, Duncan and colleagues[27] found parent-reported improvements in both treatment groups. While only a marginal number of parents reported gains in the wait-listed group, both acupuncture and osteopathy were associated with a range of positive outcomes, including arm/leg use, restful sleep, bowel function, and mood. It appears that both osteopathy and acupuncture are equally effective in reducing the limitations of cerebral palsy in children, although conclusions are limited given the lack of inferential statistics. A further limitation is reliance on parents' (unblinded) reports.

**TABLE 5**
**Neurological Disorders**

| Study | Design | Group Type | Subjects | # of Tx. | Outcome Measures | Results |
|---|---|---|---|---|---|---|
| Svendberg<br>2001<br>(Sweden) | Convenience<br>Single subject design | Acupuncture and e-stim. | n = 6<br>4–16 years<br>83% female | First course: 7–8 tx. (20 min) biweekly<br>Second course: 5–8 tx. (20 min) biweekly | Changes in hand/skin temp. | ↑Hand temp. in three subjects<br>↑Feet temp. in one subject<br>Cumulative ↑temp. for two subjects |
| Wong<br>2001<br>(Hong Kong) | Convenience | Tongue acupuncture | n = 10<br>2–18 years<br>50% female | Daily tx 5 days/week for 6 weeks | Drooling:<br>VAS<br>Quotient<br>Severity and frequency | Improvement on all three measures<br>6-Month follow-up: mother reported improvement |
| Duncan<br>2004<br>(USA) | RCT<br>Qualitative | Acupuncture<br>Osteopathy<br>Wait-list controls | n = 19<br>n = 23<br>n = 19<br>1–12 years<br>24% female | 24 weeks (unspecified dose) | Parent reported perceptions of child's cerebral palsy fx | 11% wait-list parents reported gains; 96% of tx parents reported gains |
| Sun<br>2004<br>(Hong Kong) | RCT<br>Double blind | Tongue acupuncture<br>Sham acupuncture | n = 22<br>n = 11<br>3–16 years<br>52% female | 40 (15 sec) sessions daily over 8 weeks | Change in:<br>Gross motor<br>Self-care<br>Social and cognitive fx. | ↑Gross motor function, self-care, and mobility in treatment group<br>↑Gross motor function in sham |

Tongue acupuncture (TAC) was used in two studies of children with neurological disorders. TAC was invented by Dr. J.G. Sun, an investigator in both studies, who posits that there are at least 40 acupuncture points on the tongue that are linked through standard body acupuncture meridians. The first TAC study[28] used a convenience sample of children with neurological disabilities and severe drooling problems who failed standard treatment. Significant improvement was reported on measures of drooling, with effects increasing over the course of treatment. A 6-month follow-up revealed continued improvement of drooling in most children, except during febrile illnesses. The second study[29] explored whether children with cerebral palsy gained improved functional ability with TAC. The control group received sham TAC, in which needles were pressed at nonacupuncture points on the tongue. After a 2-week wash-out period, the control group received a course of real TAC. The primary outcome measure was changes in gross motor function using a standardized scale. Secondary outcome measures included self-care and social and cognitive function using a pediatric evaluation and cognitive function index. The treatment group showed improvements in motor functioning, self-care, and mobility, while the sham group only showed improved motor functioning. The authors argue that the similar gross motor functioning between the groups may have been due to increased ambulation that both the true and sham acupuncture afforded.

Both TAC reports stated that there was initial crying with fear and possible pain for the participants during the first few sessions. No other side effects were mentioned. However, no attrition rate information was reported, making the acceptability of the technique difficult to comment upon. Before advocating the general use of TAC, studies using body points should be considered that may be less traumatic for children, especially those with disabilities. Generally, the findings relating to disabilities may be problematic due to small sample sizes. In addition, these studies lack randomization, control groups, and blinding to group status.

## Miscellaneous Studies

The remaining studies (see Table 6) include stress in premature infants, asthma, allergic rhinitis, and constipation. A pilot study explored whether electrical stimulation to acupuncture points reduced salivary cortisol levels during an ophthalmologic examination in infants[30]. Subjects were randomized to receive true electrical transcutaneous stimulation or sham (current off) treatment. Control infants displayed significant changes in cortisol, whereas cortisol levels remained statistically unchanged in the acupuncture group. The results suggest a blunting of the salivary cortisol stress response for patients receiving real electrical stimulation. The small sample size limits generalizability, but the study does point to the potential of acupuncture for decreasing stress in premature infants and warrants a larger study.

**TABLE 6**
**Miscellaneous Studies**

| Study | Design | Group Type | Subjects | # of Tx. | Outcome Measures | Results |
|---|---|---|---|---|---|---|
| Schwartz<br>1998<br>(USA) | RCT<br>Single blind (stress in prems) | E-stim.<br>Control | n = 7<br>n = 9<br><34 months<br>45% female | Single tx. during ROP exam<br>Control with e-stim. off | Salivary cortisol levels | Significant ↑ in cortisol only in control group |
| Broide<br>2001<br>(Israel) | Convenience (constipa-tion) | Crossover:<br>Acupuncture<br>Sham<br>Normal controls | n = 17<br>n = 15<br>3–13 years<br>29% female | Five weekly placebo tx.<br>Followed by ten weekly acupunc-ture tx.<br>Controls -no tx | Frequency of bowel movements (BM)<br>Panopioid blood | Max. ↑ in BM after five true acupuncture tx. for females.<br>Gradual ↑ during acupuncture for boys. Max. ↑ after 10 real tx. |
| Gruber<br>2002<br>(Austria) | RCT<br>Double blind (asthma) | Crossover:<br>Laser acupuncture<br>Sham | n = 44<br>7–16 years<br>45% female | Single laser acupunc-ture treatment | Pulmonary function tests | No significant difference between acupuncture vs. placebo |
| Ng<br>2004<br>(Hong Kong) | RCT<br>Double-blind (rhinitis) | Acupuncture<br>Sham | n = 35<br>n = 37<br>6–20 years<br>65% female | Biweekly for 8 weeks | Daily rhinitis, VAS scores, meds, blood eosinophil, serum IgE | ↓Rhinitis scores<br>↑Symptom-free days<br>↑VAS improvement scores for acupuncture |

Gruber[31] investigated the effect of a single laser acupuncture treatment in preventing a cold-dry hyperventilation-induced bronchoconstriction. All subjects had a history of bronchial and exercise-induced asthma. Following treatment, the children were exposed to a cold-dry air challenge. Pulmonary function studies were performed prior to and after acupuncture and again at 3 and 15 min after the cold-dry air challenges. Results revealed no significant differences between real and placebo acupuncture. The authors did not specify what constituted placebo acupuncture, and only indicated that the same technique was used for real and placebo treatments. It is also unclear whether the use of a single laser treatment constitutes a sufficient course of treatment for bronchoconstriction.

A well-designed large-scale trial for persistent allergic rhinitis revealed acupuncture to be superior to a sham acupuncture control group on daily rhinitis scores, ratings of overall functioning, and symptom-free days[32]. However, there were no differences between the groups on medication use or biological markers, including blood eosinophil counts, serum IgE levels, and nasal eosinophil counts. The results suggest that true acupuncture is effective in decreasing a variety of symptom scores for allergic rhinitis in children, although the benefits do not extend to biological indices.

A study of constipated children revealed sex differences in acupuncture efficacy[33]. Children first received placebo acupuncture (needle inserted into stratum corneum level at nonacupuncture points), followed by true acupuncture. At baseline, basal panopioid levels were lower in all constipated children compared to normal controls, with a gradual increase up to 73.8% in the treatment group after 10 true acupuncture treatments. Girls' bowel movements increased after the placebo treatments followed by maximal improvement after the first five of 10 true acupuncture treatments. Boys showed slower improvement. Although the findings suggest the use of acupuncture to treat constipation may be promising, larger well-designed studies are needed to replicate these findings. It should also be noted that 10 of the 27 children recruited for the study were excluded prior to the true acupuncture treatments because of poor compliance, indicating that the acceptability of acupuncture to parents and children needs further exploration.

The findings of this miscellaneous group of studies were limited by similar methodological problems found in many of the other studies reviewed. Small sample sizes, unclear design, and inconsistent definition of treatments that comprise acupuncture are common. Although it is difficult to make comparisons between the studies reviewed as "miscellaneous", the findings nevertheless indicate that acupuncture may be beneficial for a number of childhood problems. In addition, sex differences in responsivity to acupuncture may prove to be a fruitful area of research.

## SUMMARY

Based on our review of the literature, acupuncture has been used for common pediatric problems, such as PONV and nocturnal enuresis. Innovative approaches have included decreasing stress in premature infants. The number of acupuncture studies for pediatric pain complaints is surprisingly limited given the many sources of acute and chronic pain affecting children, from standard infant procedures, such as circumcision, to postoperative pain management and a range of chronic pain conditions.

There were several variations of acupuncture used in the pediatric studies reviewed above, including use of needles, electrostimulation, bleeding, tongue points, and intradermal needles with acupressure. Other studies chose to not use needles, but instead applied pressure, electricity, or laser to acupuncture points. The decision not to use needles may be an attempt to reduce children's anxiety. A further effort to reduce children's anxiety may involve combining treatments. For example, Zeltzer and colleagues[4] successfully used acupuncture with hypnosis to reduce anxiety and pain in children. As it is unknown which acupuncture modality is most beneficial, RCTs are needed to assess the efficacy of each variation. Other limitations include small sample sizes, nonvalidated measures or poorly described outcomes, and methodology problems including lack of randomized, controlled designs. In addition, the use of sham acupuncture by inserting needles into "nonactive" points has been questioned. It is possible to unintentionally activate a meridian, resulting in unanticipated benefits that invalidate the use of this approach. Future research must be mindful of sham placebos that do not tap into legitimate acupuncture meridians. Despite these issues, the efficacy of acupuncture for a variety of childhood conditions seems quite promising.

## RECOMMENDATIONS

Most studies examined the extent of adverse side effects from using acupuncture. The only reported adverse effects were found in the studies using tongue acupuncture[28,29], where children initially were fearful and cried. The consensus among the research reviewed is that there were no lasting adverse side effects from use of acupuncture, although further large-scale, well-designed studies are needed before definitive statements can be made regarding the safety of acupuncture. It has been suggested that acupuncture may have low acceptability because many children are fearful of needles, however, at least two separate studies found a high level of acupuncture acceptance in children[1,4]. Further research designed specifically to assess parent and child beliefs about the acceptability of acupuncture is required to gauge how well acupuncture would be received to treat a variety of childhood problems. One recent study found that a group of parents and children attending a pediatric pain clinic rated acupuncture as among the lowest in a list of CAM interventions, believing it not to be particularly helpful[34]. Should acupuncture emerge as a safe, viable treatment equal or superior to standard care, steps may need to be taken to educate the community regarding its use and efficacy in children.

Overall, pediatric acupuncture research studies with larger sample sizes are needed to validate the findings of existing studies. With the exception of the nausea and vomiting research, most of the pediatric acupuncture research is limited. With such sparse research being conducted in pediatric acupuncture, all studies should be encouraged, but larger studies will ultimately be most valuable. Chronic pain is reported to affect 15–20% of children[35], and more research needs to be funded and published in the field of pediatric pain management. Use of surveys to determine why parents take their children to acupuncturists may further inform future research topics. Overall, there is a lack of pediatric acupuncture research being conducted. Since there is some preliminary research suggesting efficacy of pediatric acupuncture for a wide variety of problems, an important future step is to conduct well-designed, ethical studies to enhance the evidence base for pediatric acupuncture.
